# Impact of Contextual Factors and Substance Characteristics on Perspectives toward Cognitive Enhancement

**DOI:** 10.1371/journal.pone.0071452

**Published:** 2013-08-05

**Authors:** Sebastian Sattler, Cynthia Forlini, Éric Racine, Carsten Sauer

**Affiliations:** 1 Faculty of Sociology, Bielefeld University, Bielefeld, Germany; 2 Institut de recherches cliniques de Montréal, Unité de recherche en neuroéthique, Montréal, Canada; 3 McGill University, Montréal, Canada; 4 Université de Montréal, Montréal, Canada; 5 Collaborative Research Center 882, Bielefeld University, Bielefeld, Germany; The Ohio State University, United States of America

## Abstract

Enhancing cognitive performance with substances–especially prescription drugs–is a fiercely debated topic among scholars and in the media. The empirical basis for these discussions is limited, given that the actual nature of factors that influence the acceptability of and willingness to use cognitive enhancement substances remains unclear. In an online factorial survey, contextual and substance-specific characteristics of substances that improve academic performance were varied experimentally and presented to respondents. Students in four German universities rated their willingness to use and moral acceptance of different substances for cognitive enhancement. We found that the overall willingness to use performance enhancing substances is low. Most respondents considered the use of these substances as morally unacceptable. Situational influences such as peer pressure, policies concerning substance use, relative performance level of peers, but also characteristics of the substance, such as perceptions of substance safety, shape the willingness and acceptability of using a substance to enhance academic performance. Among the findings is evidence of a contagion effect meaning that the willingness was higher when the respondents have more CE drug users in their social network. We also found deterrence effects from strong side effects of using the substance, as well as from policy regulations and sanctions. Regulations might activate social norms against usage and sanctions can be seen as costly to users. Moreover, enhancement substances seem to be most tempting to low performers to catch up with others compared to high performers. By identifying contextual factors and substance characteristics influencing the willingness and acceptability of cognitive enhancers, policy approaches could consider these insights to better manage the use of such substances.

## Introduction

Cognitive enhancement (CE) refers to a set of practices aiming to augment cognitive capacities (e.g., memory, concentration) in healthy individuals, usually with the goal of improving academic or professional performance [1], [2], [3], [4]. Much of the discussion around cognitive enhancement is centered on the use of various substances. These substances can be over-the-counter (OTC) drugs (e.g., vitamin pills; guarana products); drugs that are only available in pharmacies (e.g., caffeine tablets); prescription drugs (e.g., donepezil, modafinil, methylphenidate), or illegal drugs (e.g., cocaine; ecstasy). In Germany, where the present study was conducted, drug availabilities differ compared to the US, for example. While OTC drugs can be also purchased outside of pharmacies in Germany, drugs that are only available in pharmacies can only be sold by professional personnel, without requiring a prescription.

Prevalence studies on the use of pharmaceuticals for CE have recently attracted attention from different stakeholders such as medical societies, clinicians, and bioethicists [5], [6]. These studies have suggested that a proportion of students (1.3 to 11% in mostly North American campuses) have reported using prescription stimulants to augment their studying abilities and academic performance [7], [8], [9], [10], [11]. The health risks involved in using these medications without medical supervision or indication are not well-characterized and have generated concerns among ethicists and scientists [6], [12], [13]. There is some evidence of other substances that are used for CE such as cocaine, ecstasy, or amphetamine [9], [10], [14]. Caffeinated substances such caffeine tablets or caffeinated drinks are well-known means of CE [7], [9], [15]. Some authors describe caffeine and other OTC drugs such as vitamin pills as “soft-enhancers” and found a prevalence of 5% among German students [10]. A combined measure of pharmaceuticals, illicit drugs, and caffeine tablets revealed a prevalence of 20% also among German students aiming to enhance cognitive performance [16]. A similar rate (16%) was found in a small-scale study among Italian students without reporting the type of substance used [17]. However, the authors assume that a reasonable share refers to OTC drugs.

Perhaps of most concern are the effects of different factors within academic environments such as the pressure to succeed in competitive environments, coercion from peers to use stimulants as enhancers, and the belief that medications represent efficacious means of improving academic performance that may encourage individuals to use such substances [1], [9], [11], [18], [19]. However, the evidence needed to inform and address many of the scientific and ethical concerns regarding CE is lacking. Several recent articles have underscored inconclusive support for the efficacy of medications used as cognitive enhancers [1], [6], [12], [20], [21], [22], [23], [24], [25], [26]. Other studies have highlighted a persistent knowledge gap with respect to general prevalence of the non-medical use of medications for enhancement [27], [28]. Further, there has been an unfortunate parallel between the prevalence of substance use and actual desirability of cognitive enhancers in academic and professional environments. This relationship has led to the assumption of imminent public demand and acceptance, requiring urgent ethical and policy responses [29], [30]. Academic ethicists have produced fiercely opposing responses on issues such as fairness and on the types of mechanisms that are needed to regulate enhancement substance use [12], [31]. Still, the reactions of students toward regulations concerning enhancement substance use in universities e.g., in test-taking situations or for other academic purposes remain to be elucidated. Furthermore, little is generally known about the influence of contextual factors and characteristics of substances on decision-making process of individuals considering the non-medical use of prescription medication for CE [12], [26], [32], [33], [34], [35], [36]. Thus, the search for efficient policies cannot be based only on well-documented mechanisms behind substance consumption.

Given the importance attributed to notions of demand and prevalence in the literature in the debate surrounding CE, we aimed to empirically examine the willingness to use CE substances among students and the general acceptability of their usage. We were also interested in assessing the factors which could influence moral acceptability and willingness to engage in CE. Several potential factors have been identified and were further investigated in this study: a) impact of different types of substances; b) levels of probability and severity of side effects [11], [33], [36], [37]; c) current performance level prior to substance use [11], [34], [38], [39]; d) perceived peer prevalence [2], [10], [18], [36], [40]; and e) influence of policies regarding substance use [6]. We believe that additional empirical data on these factors could assist ethicists, clinicians, and scientists in navigating this debate and clarify their responsibility to respond to a perceived acceptance of CE of the general public and patients.

## Methods

### Sample

We employed a three-stage random selection procedure to sample university students from four universities in different academic disciplines. 2,127 students were invited to participate in this self-administered web survey. In all, 1,852 students responded (87.1%; completion rate 83.0%). 61.4% of the respondents were female. Due to dropouts and item non-response, the analyses are based on a reduced sample size (1,742 for willingness and 1,743 for acceptability).

Partnering universities informed their students of the study via post one week prior to the study in an effort to increase the awareness of the subsequent email they would receive and to motivate invitees to respond [41], [42]. After one week, all students received an email from their universities containing a personal link to the survey, as well as up to two reminder mails. Students could not fill out more than one survey. In order to achieve a higher participation rate and higher data quality, participants of the study received incentives with a value of EUR 5, e.g., [43]. We offered five options at the end of the survey: money could be sent by mail, sent to a PayPal account, replaced with a voucher for a popular online retailer, or donated to UNICEF or Amnesty International.

### Experimental Setting

We used a factorial survey–an experimental tool to investigate causal effects in survey research–that consisted of descriptions, so called vignettes [44], [45], [46], [47], [48], [49], of substances that can enhance academic performance. The vignettes provided information on substance characteristics (type of substance, probability and severity of side effects) and contextual factors (relative performance, peer prevalence and policy). Each vignette consisted of 6 dimensions with three or four levels (see Table 1). An example of a vignette would read (text in square brackets indicates experimentally varied information):

**Table 1:** Vignette Dimensions and Levels used in this study.

Dimension	Levels
1) Substance	A university student, who has an important examination soon, learns about
	the following substances that improve academic performance:
	- an over-the-counter drug.
	- a drug that is only available in pharmacies.
	- a prescription drug.
	- an illegal drug.
2) Severity of side effects	Furthermore, by taking the substance…
	- mild side effects are possible (e.g. slight restlessness or light headache).
	- moderate side effects are possible (e.g. anxiety or nausea).
	- severe side effects are possible (e.g. psychosis or heart attack).
3) Probability of side effects	These can occur in one of…
	- 10,000 users
	- 100 users
	- 10 users
4) Performance	In terms of performance, the student is…
	- among the worst 5% of students in his age-group.
	- average for his age-group.
	- among the best 5% of students in his age-group.
5) Peer prevalence	And substances like these are used by…
	- none of his fellow students.
	- half of his fellow students.
	- all of his fellow students.
6) Policy	The examination rules of his university…
	- do not forbid the use of such substances to improve academic performance.
	- strictly forbid the use of such substances to improve academic performance but do not penalize students for doing so.
	- strictly forbid the use of such substances to improve academic performance upon penalty of failing the exam.

Table 1 shows the varied dimensions of our vignettes and their levels. The order of dimensions in our study was: 1, 4, 5, 6, 2, and 3. To ease the presentation of results in this article, we have changed the order and first present all substance properties (1, 2, 3), followed by all contextual factors (4, 5, 6).

“A university student, who has an important examination soon, learns about the following substances that improve academic performance: [*a prescription drug*]. In terms of performance, the student is [*among the worst 5% of students in his age-group*]. And substances like these are used by [*half of his fellow students*]. The examination rules of his university [*strictly forbid the use of such substances to improve academic performance but do not penalize students for doing so*]. Furthermore, by taking the substance [*mild side effects are possible (e.g. slight restlessness or light headache)*]. These can occur in one of [*10,000*] users.”

In total, our experimental setup contained 972 different vignettes (combinations of factors). We applied a full factorial design in which all vignettes were used and where a single vignette was randomly assigned to each respondent. The vignette universe also contained some extreme and rarely existing situations such as the combination of OTC drugs with harmful side effects. This was done to test how individuals would decide in theoretically interesting, but rare, hypothetical, or future scenarios. We introduced the hypothetical character of all vignettes by the opening paragraph: “please imagine the following situation” signaling that students should put themselves in this situation and imagine their reaction. None of the presented combinations were logically impossible or unimaginable. Scholars have debated whether extreme vignettes should be omitted in order to provide respondents with more realistic scenarios or whether the vignettes should be investigated and therefore allow scholars to learn from these cases as well as to uphold the orthogonal design of their experiment [50], [51], [52], [53], [54]. In this vignette study, we have opted to preserve the extreme vignettes. Testing theoretically interesting, hypothetical, or futuristic scenarios is one of the advantages of vignette-based investigations. From the literature, we know that OTC drugs can also be harmful. For example, drugs containing ephedrine had some severe side effects such as severe heart valve abnormalities [55], [56], [57]. Another report also provides evidence for abuse and addiction from the use of OTC amphetamine-like stimulants containing ephedrine, pseudoephedrine, and phenylpropanolamine [58]. Even energy drinks can be risky causing unexpected deaths, as shown in warnings from the Food and Drug Administration (FDA) [59]. To assess the impact of including extreme cases in our results, we ran several additional analyses (available upon request) to test the effect of excluding extreme vignettes. No significant changes were found. This high stability of the models and effect sizes can be understood as additional indicators for the validity of the responses. Furthermore, we had very low item non-response, which can be seen as an additional indicator that responding to our vignettes was not problematic for participants.

In the experimental setup each participant evaluated one vignette. We chose vignettes for the given research question because their hypothetical character reduces the tendency to provide socially desirable answers compared to direct questioning [60], [61], [62].

### Dependent Variables

#### Willingness to Use a Substance

Participants were asked to answer the question: “Please imagine the following situation: If you were this student, would you take this substance?” We used a 10-point Likert-scale ranging from “under no circumstances” (0) to “in every case” (9). Eight participants refused to answer the question on willingness.

#### Moral acceptability

The moral perception of using a substance as described was assessed with the question: “What is your opinion about how morally acceptable it is to use this type of substance to improve academic performance?” Responses were gathered on a 10-point Likert-scale ranging from “totally unacceptable” (0) to “completely acceptable” (9). Eight participants refused to provide their judgment about moral acceptability.

One half of the respondents received the question on moral acceptability first; the other half received the question on the willingness to use the substance first. We used this experimental split to control for a question order effect. No influence of question order was found for the willingness and the moral acceptability measure.

### Ethics Statement

In Germany, research studies in social science research are not required to undergo formal ethics review. Formal ethics review is only mandatory when the research objectives refer to issues which are regulated on a legal basis (for instance in the German Medicine Act (Arzneimittelgesetz, AMG), in the Medical Devices Act (Medizinproduktegesetz, MGP), in the Stem Cell Research Act (Stammzellenforschungsgesetz, StFG), or through the Medical Association's Professional Code of Conduct (Berufsordnung der Ärzte)). Given that none of the objectives of the present study fall in the aforementioned categories, we were not obliged to seek formal ethics approval. Our research design was guided by and adheres to the ethical principles of the WMA Declaration of Helsinki. The Data Protection Act of North Rhine Westphalia (Datenschutzgesetz Nordrhein-Westfalen - DSG NRW, paragraph 28), stipulates that personal data should be anonymously processed. Only in the case of coded or nominal data usage is consent of participants is required. Our research design was endorsed by the legal services of Bielefeld University. We employed several measures to ensure the wellbeing and privacy of study participants. We applied a fully anonymous survey design in which we never accessed personal data of respondents such as names, email or postal addresses we never accessed by us. Partnering universities, which contacted the students, had no access to survey data. Online responses were protected via secure sockets layer protocols (SSL). The use of these mechanisms in addition to our methods for data handling, data usage, and deletion of data as well as the voluntariness of participation in this research were communicated to all participants in disclosure statements on the first page of the questionnaire and prior to participation (i.e., pre-notification letter, a declaration of data security, and emails). As we informed participants of conditions of the study, we interpreted the act filling out the questionnaire as implied consent. An official data protection officer supervised our project and data collection. The data of this study are available from the first author upon request.

### Statistical Analysis

We applied multivariate negative binomial regression models [63] to investigate how willingness and moral acceptability vary with (1) different types of substances associated with a range of probability and severity of side effects, and (2) within a context where students are at different performance levels, having more CE drug users in their social network, and subject to an absence/presence of policies regarding substance use. This class of models is appropriate for right-skewed distributions; it takes unobserved heterogeneity among observations into account, and helps to get more efficient, consistent and less biased estimates [64]. In our analyses, we report effects as significant if they are below an α level of 5%. Incidence rate ratios (IRR) above 1 indicate positive effects, while those below 1 indicate negative effects and those equal 1 reveal no effect.

## Results

### Willingness to use a performance enhancing substance

**Figure 1:**
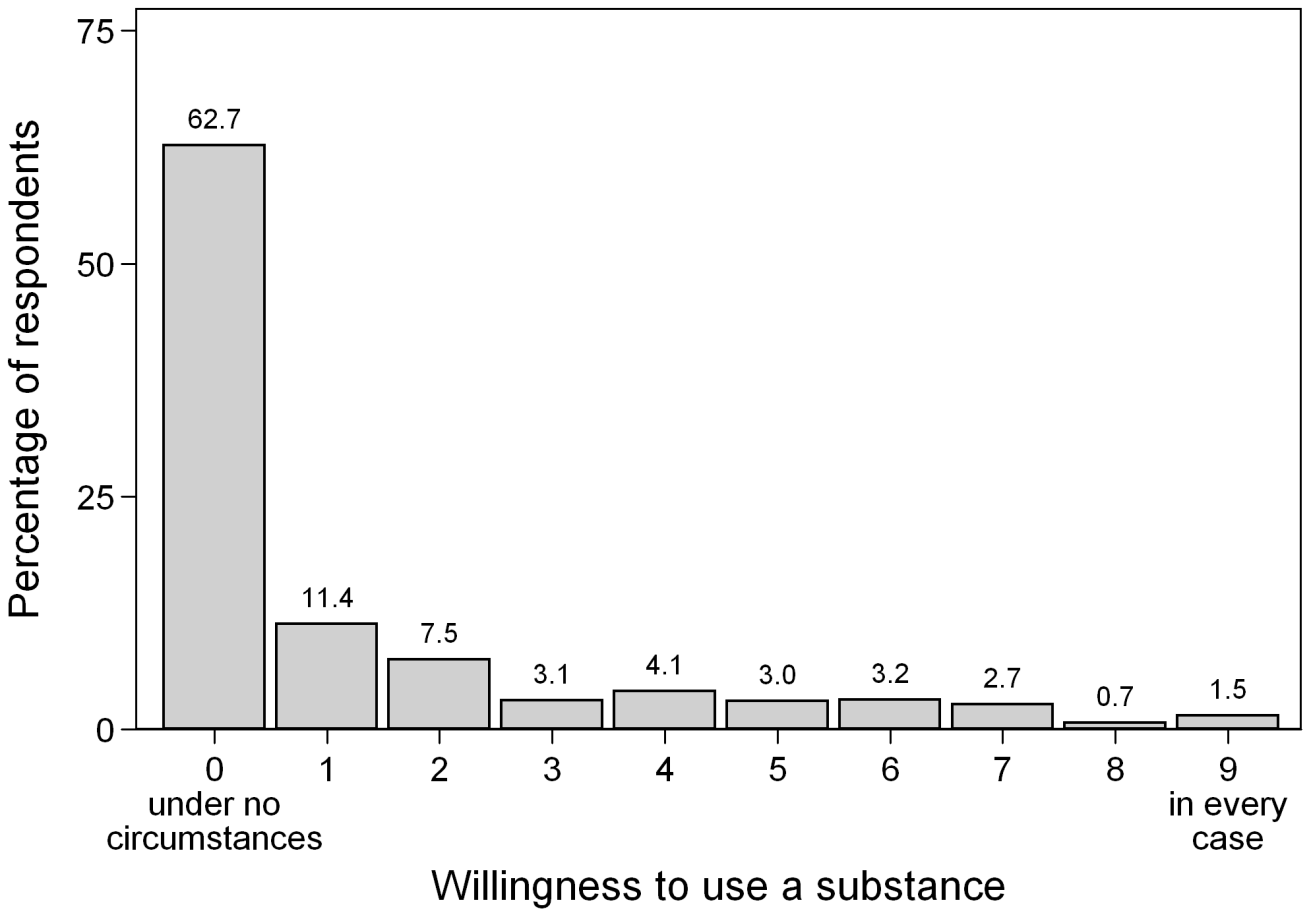
Mean Willingness to Use a Substance for Enhancing Cognitive Performance. Measured on a 10-point Likert-scale with anchors: 0: “under no circumstances”; 9: “in every case” (n  =  1,742).

According to our vignettes, the overall willingness to use performance enhancing substances was low (see Figure 1). Almost 2 out of 3 (62.7%) participants stated that they would under no circumstances decide to use a substance as described in the vignette. Only a very small amount of students (1.5%) said they would definitely use a substance for cognitive enhancement in every case.

**Figure 2:**
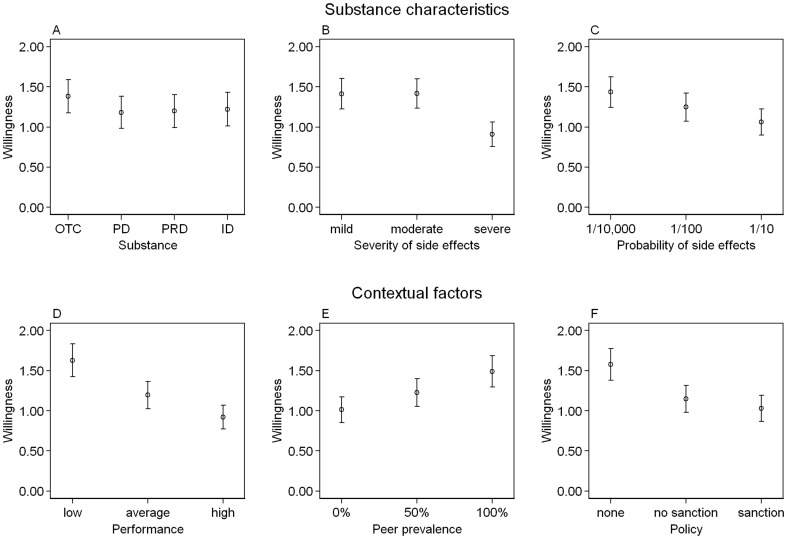
Willingness to Use a Substance with Varying Characteristics and Under Differing Contextual Factors. In each panel: Mean willingness to use a substance on a 10-point Likert-scale with anchors: 0: “under no circumstances”; 9: “in every case” and corresponding error bars. Panels A to C focus on the substance characteristics: (A) different types substances including an over-the-counter drug (OTC), pharmacy drug (PD), prescription drug (PRD), and illegal drug (ID); (B) varying severity of side effects (mild, moderate, severe); and (C) differing probabilities that side effects will occur (1 in 10.000, 1 in 100, 1 in 10). Panels D to F focus on varying contextual factors: (D) differing performance level relative to peers (low, average, high); (E) prevalence of use among peers (0%, 50%, 100%); and (F) presence or absence of policy and sanction (no policy, policy without sanctions, policy with sanction). (n  =  1,742; refer to Table 1 for the full description of the vignette levels).

Applying a negative binomial regression model (see Model 1 in Table 2), no significant differences in the decision outcome were found when different types of substances were offered, be it an OTC drug, a drug only available in pharmacies, a prescription drug, or an illegal drug (see also Figure 2). Severe side effects (e.g., psychosis or heart attack) caused by the substance deterred the willingness to enhance performance compared to mild (e.g., slight restlessness or light headache; p<0.001) or moderate (e.g., anxiety or nausea; p<0.001) side effects. An IRR of 0.638 indicates that the mean willingness decreases by a factor of 0.683 or 31.7% ((1-IRR)*100) comparing a situation of moderate with severe side effects. Mild and moderate side effects (e.g., slight restlessness or light headache) produced equal answers (p  =  0.969). When side effects occurred in one of 10,000 users, the willingness to take the substance was higher than for a substance which provoked side effects in one of 10 users (p  =  0.005). The comparisons concerning the probability of side-effects revealed no differences. If the hypothetical student belonged to the 5% lowest performing students in his class, the willingness to use the described substance was significantly higher compared to a student with an average performance (p<0.001) or to the top 5% performing students (p<0.001). The willingness was also higher, when the hypothetical student belonged to the average performance level instead of the best (p  =  0.045). The highest willingness was found when the vignette scenario stated that every fellow student used the substance. This willingness was significantly higher compared to a situation when no fellow students (p<0.001) or half of the students (p  =  0.033) used the substance. In a situation where no one used it, the willingness was also lower compared to one where half of the students used it (p  =  0.039). In a situation where the examination rules of the university strictly forbade and penalized the use of the substance, the willingness was lowest and equivalent to a situation where it was forbidden but not penalized (p  =  0.412). When there were no policies, students were much more willing to use the substance compared to a policy with (p<0.001) or without penalties (p  =  0.001). In Model 2, we added control variables and found that gender (p  =  0.523) and age (p  =  0.348) had no impact on the evaluation of the vignettes. We used the results from these models to gain insight on the most extreme vignettes (those resulting in the empirically highest and the lowest willingness). We combined the most attractive attributes of a vignette concerning social context and characteristics of substances (an OTC drug with mild side effects in 1 of 10,000 users in a university with no policy, where one of the worst 5% is surrounded by fellows who all take this substance). This analysis revealed a relatively high predicted willingness to use the substance offered (3.60), while it was very low for a more regulated substance and less attractive context (0.35; an illegal substance with severe side effects in 1 of 10 users in a university in which the substance is forbidden and penalized and where one of the best 5% students is surrounded by fellows who do not take this substance). This result indicates that some combinations of substance characteristics and contextual factors are seen as attractive and conducive to responses in favor of using the drug.

**Table 2:** Negative binomial regression of willingness on vignette dimensions respondent's sex and age.

	Model 1		Model 2	
	IRR	95% CI	IRR	95% CI
**Substance**				
over-the-counter drug (OTC = Ref.)				
pharmacy drug (PD)	0.874	[0.694–1.100]	0.872	[0.692–1.099]
prescription drug (PRD)	0.885	[0.701–1.117]	0.886	[0.702–1.117]
illegal drug (ID)	0.828	[0.662–1.035]	0.826	[0.660–1.033]
**Severity of side effects**				
mild (Ref.)				
moderate	1.004	[0.830–1.214]	0.999	[0.825–1.209]
severe	0.638***	[0.515–0.791]	0.638***	[0.515–0.790]
**Probability of side effects**				
1/10,000 (Ref.)				
1/100	0.857	[0.703–1.045]	0.862	[0.708–1.051]
1/10	0.746**	[0.607–0.917]	0.747**	[0.608–0.917]
**Performance**				
low (Ref.)				
average	0.692***	[0.569–0.841]	0.696***	[0.572–0.847]
high	0.555***	[0.452–0.682]	0.554***	[0.451–0.680]
**Prevalence**				
0% (Ref.)				
50%	1.248*	[1.012–1.539]	1.243*	[1.009–1.532]
100%	1.545***	[1.257–1.901]	1.538***	[1.251–1.891]
**Policy**				
none (Ref.)				
no sanction	0.712***	[0.587–0.862]	0.715***	[0.590–0.866]
sanction	0.650***	[0.529–0.798]	0.650***	[0.529–0.798]
**Controls**				
male (Ref. = female)			1.058	[0.890–1.258]
age			0.976	[0.928–1.027]
				
Constant	2.469***	[1.853–3.291]	2.684***	[1.865–3.862]
Chi^2^	100.65***		101.41***	
Number of Vignette Evaluations	1,742		1,742	

*p<0.05,

**p<0.01,

***p<0.001 (robust standard errors).

Table 2 shows the incidence rate ratios (IRR) and confidence intervals (CI) in parentheses of the willingness to use a CE substance on six vignette dimensions (Model 1) and additionally adjusted for gender and age in Model 2.

### Moral Acceptability of Using a Performance Enhancing Substance

**Figure 3:**
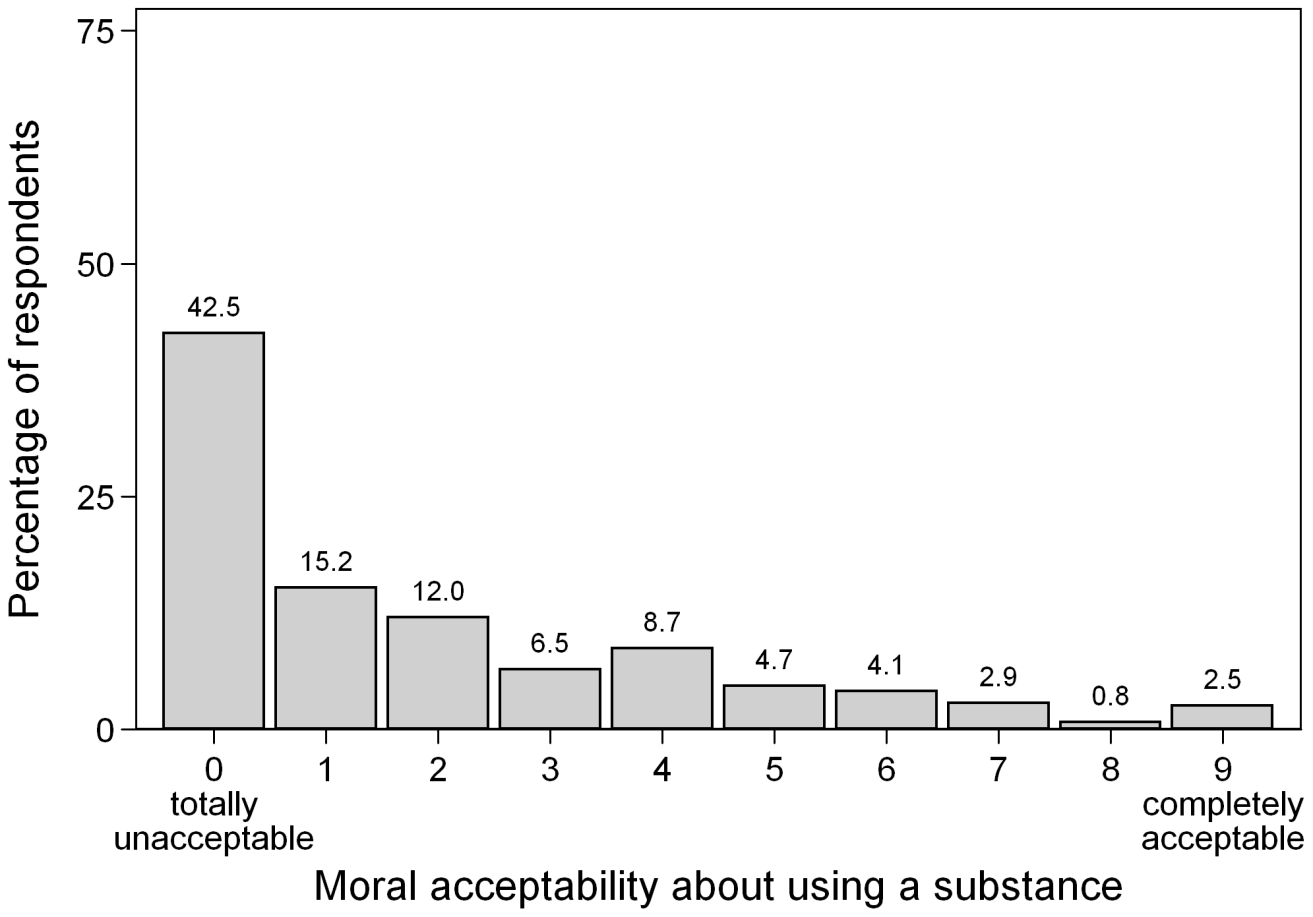
Mean Moral Acceptability about Using a Substance for Enhancing Cognitive Performance. Measured on a 10-point Likert-scale with anchors: 0: “totally unacceptable”; 9: “completely acceptable” (n  =  1,743).

A substantial minority (42.5%) of participants considered substance use, as described in the vignettes, as morally unacceptable and only a few (2.5%) found it completely acceptable (see Figure 3).

**Figure 4:**
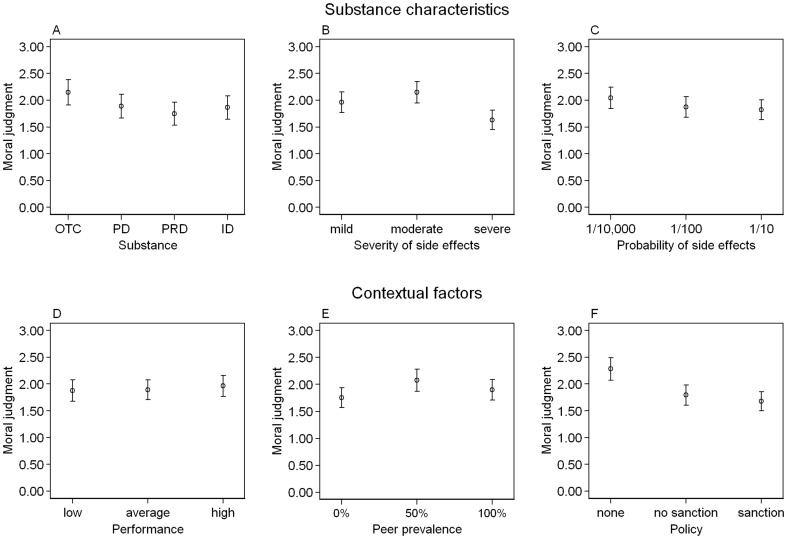
Moral Acceptability of Using a Substance with Varying Characteristics and Differing Contextual Factors. In each panel: Mean moral acceptability about the use of a substance on a 10-point Likert-scale with anchors: 0: “totally unacceptable”; 9: “completely acceptable” and corresponding error bars. Panels A to C focus on the substance characteristics: (A) different types substances including an over-the-counter drug (OTC), pharmacy drug (PD), prescription drug (PRD), and illegal drug (ID); (B) varying severity of side effects (mild, moderate, severe); and (C) differing probabilities that side effects will occur (1 in 10.000, 1 in 100, 1 in 10). Panels D to F focus on varying contextual factors: (D) differing performance level relative to peers (low, average, high); (E) prevalence of use among peers (0%, 50%, 100%); and (F) presence or absence of policy and sanction (no policy, policy without sanctions, policy with sanction). (n  =  1,743; refer to Table 1 for the full description of the vignette levels).

Regression analyses showed that OTC drugs were seen as morally more acceptable than prescription drugs (p  =  0.007; see Model 1 in Table 3; see also Figure 4) and illegal drugs (p  =  0.033). No further differences were found between the types of substance used for CE. Severe side effects reduced the acceptability of substance use compared to mild (p  =  0.010) or moderate (p<0.001) side effects. Mild and moderate side effects produced equal answers (p  =  0.256). No association effect of the probability of side effects was found. We also found no impact of the performance level of the student on acceptability. Respondents considered the use of the pill as (p  =  0.025) morally more acceptable when half of the fellows used it compared to a situation when nobody used it. Further comparisons with regard to peer prevalence showed no differences. Acceptability was higher when the university did not forbid the use of the substance compared to a situation when it was forbidden (p<0.001) or forbidden and additionally punished (p<0.001). There was no difference between forbidden use with or without a penalty (p  =  0.546).

**Table 3:** Negative binomial regression of moral acceptability on vignette dimensions respondent's sex and age.

	Model 1		Model 2	
	IRR	95% CI	IRR	95% CI
**Substance**				
over-the-counter drug (OTC = Ref.)				
pharmacy drug (PD)	0.882	[0.752–1.036]	0.877	[0.747–1.028]
prescription drug (PRD)	0.797**	[0.676–0.940]	0.798**	[0.677–0.941]
illegal drug (ID)	0.840*	[0.716–0.986]	0.840*	[0.716–0.986]
**Severity of side effects**				
mild (Ref.)				
moderate	1.082	[0.944–1.240]	1.076	[0.939–1.233]
severe	0.822**	[0.709–0.953]	0.821**	[0.709–0.951]
**Probability of side effects**				
1/10,000 (Ref.)				
1/100	0.916	[0.796–1.055]	0.917	[0.797–1.054]
1/10	0.902	[0.782–1.041]	0.910	[0.790–1.050]
**Performance**				
low (Ref.)				
average	1.019	[0.884–1.175]	1.025	[0.890–1.182]
high	1.072	[0.928–1.239]	1.067	[0.924–1.233]
**Prevalence**				
0% (Ref.)				
50%	1.179*	[1.021–1.360]	1.184*	[1.027–1.365]
100%	1.087	[0.940–1.256]	1.084	[0.939–1.252]
**Policy**				
none (Ref.)				
no sanction	0.772***	[0.671–0.887]	0.771***	[0.672–0.886]
sanction	0.737***	[0.640–0.849]	0.740***	[0.643–0.851]
**Controls**				
male (Ref. = female)			1.194**	[1.060–1.345]
age			1.012	[0.977–1.049]
				
Constant	2.558***	[2.069–3.162]	2.246***	[1.724–2.927]
Chi^2^	51.47***		64.44***	
Number of Vignette Evaluations	1,743		1,743	

*p<0.05,

**p<0.01,

***p<0.001 (robust standard errors).

Table 2 shows the incidence rate ratios (IRR) and confidence intervals (CI) in parentheses of the moral acceptability to use a CE substance on six vignette dimensions (Model 1) and additionally adjusted for gender and age in Model 2.

We found that men rated the type of substance as more objectionable than women (p  =  0.004, see Model 2), but we found no effect of age (p  =  0.493). For the moral acceptability, we also investigated the most empirically extreme vignettes (as was done for willingness with slight changes for the least attractive scenario: prescription drugs instead of illegal drugs and average instead of worst). When offering the most attractive attributes of a vignette concerning social context and substance characteristics, the acceptability was 3.37, while it was 1.11 for the least attractive substance and context. Compared to similar analysis for willingness, it can be concluded that willingness has a higher elasticity with regard to situational stimuli than judgments about moral acceptability.

## Discussion

### Summary and Interpretation of Results and Reflection on Prior Research

A fierce debate has surfaced on the clinical and ethical acceptance of CE. One indication of ethics acceptance could be prevalence of use cognitive enhancing substances. However, prevalence data has been criticized on methodological grounds [65] and the actual nature of factors that encourage or discourage CE substance use among the general public or students has not been well characterized. Such information would be useful for developing empirically-grounded regulations on substances used for CE. This vignette-based study is among the first examining influences on the willingness and judgment about moral acceptability of CE substance use. Most studies have concentrated only on one or a few aspects of CE to study willingness and acceptability. Our approach combined recurring issues in the current debate on CE to present an exhaustive research design that constructed specific social contexts and complex combinations of characteristics of substances than previous studies. Our findings are relevant to the discussion of rates of acceptability of and willingness to engage in CE substance use, the factors affecting them as well as implications for public health policies.

### Low overall willingness and acceptability

We found overall very low willingness to engage in different forms of CE as well as generally unfavorable attitudes toward the moral acceptability of CE – especially when we provided less attractive contexts and substance characteristics. These results question previous statements found in highly cited commentaries [6], [12] and policy documents [66] stating the contrary. The academic debate and media tempest surrounding CE have hinged on the description of a widespread trend of misuse or non-medical use of pharmaceuticals in students [5], [8], [27], [28], [29], [34], [38]. Moreover, public demand for CE has been predicted to be significant, even to the point of making CE “inevitable” [18], [19]. Guidance from the American Academy of Neurology [66] and discussions from other medical bodies has been triggered based partly on this perception [5] and commentators have claimed the same [12], [67]. Likewise, media coverage, internationally and in particular in the US, has heralded a powerful trend of CE captured in enthusiastic discourse [68], [69]. Our findings therefore stand in contrast to prior publications and comments suggesting widespread acceptance of and willingness to engage in CE. Perhaps this contrast can be partly explained by the German context of this study, which could be less willing and accepting of CE, or a slow trend follower rather than the trend setter in this area. Other potential reasons might be differences in the legal and illegal access to enhancement substances, restricted pharmaceutical advertisement in Germany [9], pressure to succeed in universities, and culturally preferred options for success (e.g., personal effort). In support of this interpretation are recent prevalence data showing a lower trend of CE on a large German campus [7], [9], [11]. However, CE studies often focus on prescription drugs (in contrast to OTC drugs), which might partially explain low prevalence rates. One study [16] combining the assessment of different types of CE substances (illicit drugs, prescription drugs, and caffeine tablets) already found a 12-month prevalence of 20% among German university students. Moreover, Franke et al. [36] found a willingness to use hypothetical enhancement substances which do not cause long-term damages or addiction in 8 out of 10 high school and undergraduate students but with a much lower reported prevalence of substances used for CE. Franke et al.'s results might indicate that CE practices go beyond prescription drug use and that CE could be a forthcoming trend, e.g., if more effective substances were available, access on the Internet and black market increases, or if the need for high and long lasting performance continues. The relatively high proportion of 80% suggests that a majority of students is tempted to use enhancers if they were safe. In the absence of comparative data, this hypothesis merits further consideration. However, at the very least, important caveats need to be kept in mind in the international context of discussions about CE. Indeed, if populations like the German students seem to be recalcitrant to CE at the moment, the generalizability of the American bioethics discussion could be compromised and further attention to cultural aspects of the debate warranted. Even in Canada and Australia, countries sharing many features with the US (not including health care systems), critical opinions regarding CE have been voiced by medical authorities [70], [71] and scholars [27], [30], [65]. Hence, it is an open question if assumptions of demand have also been largely overestimated in the American context and those of culturally proximate nations. Nonetheless, dissemination of high estimates could be consequential. Focus group-based research has found that stakeholders (university students, parents, healthcare professionals) react strongly to media reporting on wide prevalence and acceptance, and internalize this trend as being inescapable and coercive CE [72].

### Distinct factors shaping willingness and acceptability

Our study design allowed us to explore the effect of various factors on willingness and acceptability. Our observations generally show that some factors discussed in the literature affect willingness slightly more than they affect acceptability but, contrary to what is sometimes implied, may not in themselves have decisive consequences on willingness and moral acceptability.

#### Legal status of drug

We found that willingness was not influenced by the legal status of the substance, but the acceptability of OTC drugs was higher compared to prescription and illegal drugs. One reason might be the differences in the access to these substances and the fact that using and dealing prescription and illegal drugs are forbidden.

#### Side effects

In line with previous research [11], [33], [36], [37], we found a lower willingness for more severe and more probable side effects. High severity, but not high probability of side effects, decreased acceptability. Given the importance attributed to side effects and potential health-risks [12], [13], it can be argued that individuals refrain from using substances having side effects because, from a behavioral perspective, such effects can be described as negative incentives or costs.

#### Normalizing and enhancement

Scholars have debated the varying acceptability for users who want to normalize their performance or those who want to enhance it [73]. However, we did not find any effect of the current performance level on moral acceptability. However, the willingness to use a substance was much higher for low performers compared to others. When considering the case of low performers, individuals might expect to gain more when using a substance [11], [34]. This might imply that current discussions fail to capture that individuals instrumentally react to the gains of using substances, but that in this case, moral reasoning is not affected by rational deliberation on benefits. Prior research also found a relationship between a low grade point average or lower competencies and an increased non-medical use of prescription medication [11], [38], [39].

#### Peer pressure

Our data show an increased willingness to use a substance but higher acceptability when 50% of peers used the substances compared to none. Also Franke et al. [36] found that a share of students would be more willing to use enhancers if others did. Generally, social networks are crucial factors in decision-making in many spheres of life by providing influential social information to individuals: they teach certain behaviors, offer support or coercion about life goals, and influence norms to be followed [18], [74], [75], [76], [77]. Consequently, a widespread use of enhancement substances has been hypothesized to encourage their use because: 1) substances might be perceived as beneficial and not risky because many people use them; 2) not using them may lead to feeling disadvantaged due to a relative lower performance [9], therefore making substance use a strategic decision; and 3) it may encourage the belief that it is not immoral to use them as CE is not deviant behavior, so normalization about its use may occur [40], [77] and students may follow a “social heuristic” [74].

#### Policy

We found a lower willingness and a lower acceptability when substance use was forbidden. Regulations not only forbidding but sanctioning substance use did not present an additional discouraging effect. The explanation of the effects found is multifaceted and will be discussed in the next section.

### Health policy implications for the use of substances for CE

One important facet in discussions about enhancement substances is regulation of their use [6]. To our knowledge, our study is the first to provide results about the effect of regulations on willingness and acceptability of CE substance use. Due to its practical impact, we therefore wish to discuss this finding and its meaning in greater detail. In the literature and in health policy, a variety of positions can be classified following a spectrum of liberal and conservative stances [78]. From a practical point of view, the impact of these regulations is of high interest. Based on our results, classifying substance use for enhancement purposes in universities as cheating might activate social norms against usage, such as the acknowledgement of the infringement of fairness. Social norms are very influential on behavior [79], [80], [81] and our results consistently support that regulations might restrict usage. Separate from our study but connected to our data is the need to develop clearer health policies about cognitive enhancement as a potential public health concern.

CE has been described as a public health concern [82] but claims to that effect have been criticized, based on rather low prevalence rates [27]. However, given the important public debate, academic discussions, and suspected higher prevalence rates on some specific American campuses, some public health authorities should consider acting to curtail CE. Our results indicate that informing potential users about the prevalence of severe side- effects could discourage the use of substances for CE. Informing them about these health risks (e.g., via drug education courses or the media) can be a promising means of prevention [9], [11], [33], [83]. Also, by simply having and promoting a policy regarding CE, academic institutions could assume a firmer and potentially more effective role in prevention strategies. This stance reflects the opinion voiced in an editorial of the *Canadian Medical Association Journal*, calling for stimulant abuse to be “recognized by universities as a life-threatening issue and then denormalized” [71]. The finding that lower performance increases willingness can be utilized by strengthening learning strategies for students who may be straggling behind. Student-focused teaching methods might also be an approach to improve a student's self-perceived competencies, but also to provide a more effective learning environment [84], [85]. We did not investigate the power of traditional strategies to improve academic performance (e.g., tutoring, counseling) which could also have a powerful impact on practices. The efficacy of these strategies compared to those of substances we studied would be worthy of investigation before policy action is taken. We did not examine the impact of broader actions (e.g., legislative) and sanctions beyond the academic context, which could also extend the effect seen in university policies. These other options merit further attention from ethicists and policy-makers and call for further research. However, our data suggest that different substances used for enhancement could be targeted collectively given similar willingness towards and acceptability of types of substances used for the specific goal of enhancement.

### Limitations of the Study

There are a few limitations to the results of our study. 1) Not all invited participants filled out the questionnaire. However, our response rate (87.1%) did reach the upper end of the rate achieved in previous studies (22 to 86%) [38]. In addition, the number of item non-response remained very low. However, the impact of nonresponse might be negligible regarding the interpretation of the results because we conducted an experiment where every student was randomly assigned to a vignette. So, all contextual factors and characteristics of substances are uncorrelated with characteristics of respondents. Finally, our results were controlled for effects of age and gender. 2) The questions regarding substance use are sensitive. Especially for non-anonymous surveys, this results in a risk of downward-biased prevalence rates [86], [87]. However, online-surveys provide a high level of anonymity and as stated in the methods section, we employed multiple measures to protect answers of respondents and to ensure high data quality (e.g., full anonymity of the survey and supervision of an official data protection officer). Furthermore, it is known that the hypothetical character of factorial survey designs is more immune to skewing answers towards social desirability than direct questioning [60], [61], [62]. Moreover, the low item non-response rate for these questions can be indicative of a low perceived sensitivity of the question and a high perceived confidentiality of answers. We also tested whether the respondents' perceived anonymity of this survey had an impact on the willingness and the acceptability measure but found no effects (results are available upon request). 3) We surveyed students only. The general population might react differently to our vignettes due to different needs and preferences. However, students are a population at risk of using enhancers because of pressures to succeed and the valuing of cognitive performance. Therefore, they might belong to a group of “early adopters” [12]. Nevertheless, replicating our study with the general population might reveal interesting insights. 4) We analyzed the willingness and acceptability in one country. As discussed above, results in different countries might differ. Though, multi-country studies would be helpful to get more insights and to compare the impact of contextual factors and substance characteristics. 5) Due to a lack of statistical power, we were not able to experimentally vary and investigate all potential influences in our study. It would be important to know more about the impact of other factors (such as the price of getting relevant substances, access, or effectiveness). However, we used an experimental approach, so the impact of potential confounders was reduced.

### Conclusion

Using an online factorial survey design with vignettes, we investigated the moral acceptability of and willingness to use enhancement substances. In spite of claims to the contrary in the literature, we found limited willingness to engage in, and moral acceptability of enhancement using substances like prescription and illicit drugs. Several factors (types of substances, probability and severity of side effects, different performance levels, number of CE drug users in the social network, and policies regarding substance use) influenced willingness and moral acceptability. Future research should address similar questions in an international context and extend the scope of influential dimensions, such as access to substances. Public interventions should take into consideration both the lack of general acceptance found in this study as well as factors that would deter use, such as highlighting the health risks of substance use, which could support effective health education or public health policies.
